# Non-inferiority of a hybrid outpatient rehabilitation: a randomized controlled trial (HIRE, DRKS00028770)

**DOI:** 10.1186/s44247-023-00013-4

**Published:** 2023-04-25

**Authors:** Richard Albers, Stella Lemke, Sebastian Knapp, Gert Krischak, Matthias Bethge

**Affiliations:** 1 Institute of Social Medicine and Epidemiology, University of Lübeck, Ratzeburger Allee 160, 23562 Lübeck, Germany; 2 GOREHA GmbH, Neue Schönhauser Straße 20, 10178 Berlin, Germany; 3 Zentrum Für Ambulante Rehabilitation, Spatenstraße 12, 88046 Friedrichshafen, Germany

**Keywords:** Hybrid rehabilitation, Telemedicine, Telehealth, Back pain, Randomized controlled trial, Non-inferiority study

## Abstract

**Background:**

Physiotherapeutic telerehabilitation in various musculoskeletal and internal diseases, including back pain, might be comparable to face-to-face rehabilitation or better than non-rehabilitation. In Germany, a standardized back school for patients with chronic back pain is provided in outpatient rehabilitation centers. The effectiveness of this standardized back school was shown in a randomized controlled trial in face-to-face rehabilitation. This study examines non-inferiority of a hybrid rehabilitation applying a digital version of the standardized back school against a rehabilitation applying the face-to-face back school.

**Methods/design:**

We recruit 320 patients in eight German outpatient rehabilitation centers. Patients are randomized equally to the intervention and control groups. Patients aged 18 to 65 years with back pain are included. Patients lacking a suitable private electronic device and German language skills are excluded. Both groups receive the standardized back school as part of the 3-week rehabilitation program. The control group receives the back school conventionally in face-to-face meetings within the outpatient rehabilitation center. The intervention group receives the back school online using a private electronic device. Besides the back school, the patients participate in rehabilitation programs according to the German rehabilitation guideline for patients with chronic back pain. Hence, the term “hybrid” rehabilitation for the intervention group is used. The back school consists of seven modules. We assess data at four time points: start of rehabilitation, end of rehabilitation, 3 months after the end of rehabilitation and, 12 months after the end of rehabilitation. The primary outcome is pain self-efficacy. Secondary outcomes are, amongst others, motivational self-efficacy, cognitive and behavioral pain management, and disorder and treatment knowledge. Guided interviews with patients, physicians, physiotherapists and other health experts supplement our study with qualitative data.

**Discussion/aim:**

Our randomized controlled trial aims to demonstrate non-inferiority of the online back school, compared to conventional implementation of the back school.

**Trial registration:**

German Clinical Trials Register (DRKS00028770, April 05, 2022).

**Supplementary Information:**

The online version contains supplementary material available at 10.1186/s44247-023-00013-4.

## Background

Over the last decade, the usage of technology in rehabilitation has grown exponentially, paving the way for the development of telerehabilitation and the opportunity for more flexible implementation of rehabilitation [1]. The benefits of telerehabilitation were mainly linked to better continuity of treatment when patients returned to their home environment from rehabilitation centers [2]. Especially in aftercare, telerehabilitation can help to consolidate achieved outcomes [3, 4]. In secondary prevention, telerehabilitation is beneficial to integrate therapeutic measures into the everyday life of the patient [3]. Moreover, the World Federation of Occupational Therapists has issued a statement on the use of telemedicine to improve accessibility to occupational therapy and other benefits, such as an effective support for the transition to home life [5]. Not least due to the SARS-CoV-2 pandemic, telerehabilitation was often the only form of rehabilitative care and tested worldwide [6–8]. Particularly, regions with a low supply density of rehabilitation services could profit from these interventions [8]. Since approximately 80% of those aged 14-years and older in Germany use the internet regularly, the required skills for participation in internet-based interventions are a given among the patients [9].

A survey from 2019/2020 showed that 61.3% of German adults suffered from back pain during the last 12 months. Low back pain occurred more often than neck pain in a ratio of two-to-one. Women were affected more often by low back pain (60%) and neck pain (54.9%) than men (56.4% and 36.2%). Around 15.6% of German adults develop chronic back pain (women 18.5% and men 12.4%) [10]. Due to sick leave, usage of health care services, and disability pensions, back pain is associated with high costs [11, 12].

Physical exercise can reduce pain and improve mobility, which is why it plays an important role in today’s pain rehabilitation programs [13]. However, reduced chronic low back pain observed in physical therapy does not appear to be sustained over the long term, providing an opportunity for telerehabilitation services to ensure continuity and sustainability [14].

Although a systematic review of exercise-based telemedicine in patients with chronic pain found no difference in physical activity, activities of daily living, and quality of life [15], telerehabilitation interventions have been shown to be beneficial in achieving improvements in low back pain to maintain or reduce the dropout rate through so-called “booster sessions” conducted via a mobile app [14] and video conferences [16]. In the study by Crane and colleagues, patients with chronic pain favored intermediate telerehabilitation programs that included feedback and monitoring technologies, as well as direct face-to-face counseling and exercises [17].

Whilst considerations on the implementation of telerehabilitation in Germany have so far been largely limited to rehabilitation access [18, 19] and rehabilitation aftercare [4], a current overview of systematic reviews suggests that physiotherapeutic telerehabilitation in diseases such as arthrosis, low back pain, hip and knee replacements, multiple sclerosis, and also within the framework of cardiac and pulmonary rehabilitation, can achieve as comparable or better outcomes as personal rehabilitation or non-rehabilitation [20]. Nonetheless, the included studies in the systematic reviews ranged from the years 2002 to 2016, whereas technologies have improved in the meantime [21, 22]. Newer technologies such as smartphone apps were not investigated [21] or stated to be understudied [22]. Additional differences between the studies, for example the definition and duration of back pain or delivery of the intervention, make an interpretation of the study’s findings difficult.

The study we are planning compares a hybrid rehabilitation program that implements an online back school with conventional face-to-face back school. The back school curriculum developed by the Federal German Pension Insurance [23] is the standard back school in all participating outpatient rehabilitation centers. Effectiveness of this back school was shown in a randomized control trial within standard face-to-face inpatient rehabilitation [24].

## Objectives

We hypothesize that patients receiving the hybrid rehabilitation program achieve as comparable pain self-efficacy as patients receiving the back school within a conventional rehabilitation program. Additional comparable outcomes are expected for other variables. Guided interviews explore the structure and processes of the hybrid rehabilitation program.

### Trial design

Our study is a randomized controlled trial. We recruit participants in eight outpatient rehabilitation centers. Each center provides 40 patients. In total, we recruit 320 patients. Within each rehabilitation center, participants are randomized equally to the intervention and control group. Randomization schedules were stratified for each rehabilitation center and used block randomization. Four measurement points are planned (start of rehabilitation, end of rehabilitation, 3 months after end of rehabilitation, 12 months after end of rehabilitation). We examine the non-inferiority of the hybrid rehabilitation program. Primary outcome is pain self-efficacy at the 12-month follow-up [25]. We also assess and analyze qualitative data using guided interviews with 20 patients and 18 experts. The interviews are carried out by phone.

## Methods

### Study setting

Eight centers for outpatient rehabilitation are involved in recruitment, data collection, and implementation of the rehabilitation program. All centers are located in seven different German cities (Berlin, Bielefeld, Frankfurt am Main, Jena, München, Paderborn, Regensburg). The intervention and control groups are recruited in the same rehabilitation center, but the online back school of the intervention group is used at the patients’ homes and is provided by a web-based rehabilitation provider (Caspar Health).

### Eligibility criteria

Rehabilitants aged 18 to 65 years and with back pain (ICD-10 M50 to M54, post-acute and acute rehabilitation) are included. Patients without stable internet, an appropriate electronic device for using the internet or executing app-related videos, and a suitable camera for communication are excluded. We also exclude patients not speaking German.

### Treatment

#### Intervention* group*

The intervention group receives a hybrid rehabilitation program, during which a standardized back school is delivered digitally. The standardized back school developed from the Federal German Pension Insurance consists of seven modules and is based on the health action process approach and the fear-avoidance beliefs model [24, 26]. The back school teaches knowledge about the development and maintenance of back pain, communicates a positive functional image of the back, demonstrates and practices exercises to strengthen the back, reflects how pain is mentally processed, and promotes the transfer of physical activity in everyday life. The Caspar application is used for the digital implementation of the back school. Table 1 shows a detailed overview of the back school in the intervention group, based on the Template for Intervention Description and Replication (TIDieR) checklist [27] and the TIDieR-Telehealth checklist [28].

**Table 1:** Description of the intervention group based on the TIDieR checklist and the TIDieR-Telehealth checklist

**Brief name**	Digital back school (hybrid rehabilitation)
**Why**	The back school aims to increase physical activity in everyday life, according to the health action process approach, and to change cognitive patterns towards pain that lead to pain chronicity, based on the fear-avoidance beliefs model [26]
**What (materials)**	All content of the back school is digitalized and accessible online, via app or web browser, using the Caspar application. Patients use their private electronic device to participate. A study assistant within each outpatient rehabilitation center gives oral and practical instructions on correct use of the Caspar application and hands out a booklet with further information on the program, including the individual login account of the patient. The multimedia content contains educative videos and videos on physical exercises. For interactive meetings, the electronic device must be equipped with a suitable camera. Furthermore, patients can communicate with a health care professional (HCP), in particular physiotherapists, individually through a chat box at any time during the week
**What (procedures)**	The back school consists of seven modules [23]: (1) fundamentals; (2) back health and physical activity; (3) body awareness and spine stabilization; (4) mental factors; (5) posture and movement sequences in everyday life and at work; (6) physical activity in everyday life Part 1; and (7) physical activity in everyday life Part 2
**Who provided**	Caspar Health is a private company that provides all features of the Caspar application. The Tele-Therapie Klinik Berlin is a telemedicine clinic and provides all HCPs that guide the interactive sessions in the intervention group online, including the chat. These HCPs are experienced and specialized in digital therapy. The Tele-Therapie Klinik Berlin plans, organizes, and schedules all the interactive meetings
**How**	The entire rehabilitation program is implemented online, using the Caspar application. Group interactive meetings are performed by an HCP via camera. Non-interactive parts of the modules are performed by each patient independently
**Where**	The patients participate in the intervention from home. The HCPs operate from the buildings of Tele-Therapie Klinik Berlin or from home
**When and how much**	The seven modules are completed within 3 weeks. Each module requires 45 min to complete. The patients can choose freely when to use the educational videos and the videos on physical exercises during the week. In addition, every week a 45-min live interactive meeting is conducted online via camera and a video conference tool. Interactive meetings are scheduled during the afternoon. The participants can choose from different time slots. In addition to the online back school, patients follow their individual 3-week rehabilitation program in the outpatient rehabilitation centers. Treatments in the rehabilitation programs follow the therapy standards developed by the Federal German Pension Insurance for the rehabilitation of chronic back pain [29]
**Tailoring**	No specific tailoring of the intervention to the patient is planned. Nevertheless, during the weekly interactive meeting and in the chat, patients can receive individual feedback from the physiotherapist and apply it accordingly (e.g., change a physical exercise if painful)
**Modifications**	Not applicable
**How well**	No strategies are implemented to maintain adherence to rehabilitation. However, it is registered if and how often the patients watched the videos and if patients joined the weekly interactive meetings. Therapy adherence is measured and assessed by the researchers at the University of Lübeck

#### Control group

The control group receives a conventional rehabilitation program, during which the standardized back school is provided in face-to-face meetings. Table 2 shows a detailed overview of the back school in the control group, based on the Template for Intervention Description and Replication (TIDieR) checklist [27] and the TIDieR-Telehealth checklist [28].

**Table 2:** Description of the control group based on the TIDieR checklist and the TIDieR-Telehealth checklist

**Brief name**	Face-to-face back school (conventional rehabilitation)
**Why**	The back school aims to increase physical activity in everyday life, according to the health action process approach, and to change cognitive patterns towards pain which lead to pain chronicity, based on the fear-avoidance beliefs model [26]
**What (materials)**	Several materials are used during the back school, such as a patient information booklet, LCD projector, and presentation sheets. A detailed list of required materials can be found elsewhere [23]
**What (procedures)**	The back school consists of seven modules [23]: (1) fundamentals; (2) back health and physical activity; (3) body awareness and spine stabilization; (4) mental factors; (5) posture and movement sequences in everyday life and at work; (6) physical activity in everyday life Part 1; and (7) physical activity in everyday life Part 2
**Who provided**	All outpatient rehabilitation centers are members of the Nanz Medico GmbH & Co. KG group. Each outpatient rehabilitation center provides their own experts from various fields (e.g., physicians, physiotherapists, or psychologists) to implement the back school
**How**	All interventions are carried out in groups within the outpatient rehabilitation centers via face-to-face meetings. Outside of the meetings, patients can make use of their materials, such as the patient information booklet, in order to practice or do the exercises
**Where**	All meetings take place in the outpatient rehabilitation center
**When and how much**	The seven modules are completed within 3 weeks. Each module requires 60 min to complete. In addition to the back school, the patients follow their individual 3-week rehabilitation program in the outpatient rehabilitation center. The treatments in the rehabilitation programs are in accordance with the therapy standards developed by the Federal German Pension Insurance for the rehabilitation of chronic back pain [29]
**Tailoring**	No specific tailoring of the intervention to the patients is planned. Nevertheless, during the meetings patients can receive individual feedback from the performing medical expert and apply it accordingly (e.g., change a physical exercise if painful)
**Modifications**	As all included outpatient rehabilitation centers are separate institutions, slight variations in performing the standardized back school may occur
**How well**	No strategies are implemented to maintain adherence to rehabilitation. However, it will be registered if patients joined each module or not. Therapy adherence will be measured and assessed by the researchers of the University of Lübeck

### Ancillary and post-trial care

No harm due to study participation is expected. Therefore, no ancillary and post-trial care are intended.

### Outcomes

All outcomes, including measurement points and expected scaling, are shown in Table 3. Since no adverse events are expected, no plans for collecting, assessing, reporting, or managing of adverse events were made.

**Table 3:** Measures, assessment, expected scaling, and measurement occasions

Outcome	Source and reference	Scaling	Scoring	Measurement time points			
				**Start of rehabilitation**	**End of rehabilitation**	**3 months follow-up**	**12 months follow-up**
**Primary outcome**							
Pain self-efficacy	FESS [25]	Continuous	10 to 60	x	x	x	x
**Secondary outcomes**							
* Health and health-related outcomes*							
Current health status	COPSOQ-Item [30]	Continuous	0 to 10	x	x	x	x
Mental health	IRES-24 [31]	Continuous	0 to 10	x	x	x	x
Functional capacity	IRES-24 [31]	Continuous	0 to 10	x	x	x	x
Pain	IRES-24 [31]	Continuous	0 to 10	x	x	x	x
Action-oriented coping	FESV [32]	Continuous	4 to 20	x	x	x	x
Cognitive restructuring	FESV [32]	Continuous	4 to 20	x	x	x	x
Subjective coping competence	FESV [32]	Continuous	4 to 20	x	x	x	x
Mental distraction	FESV [32]	Continuous	4 to 20	x	x	x	x
Counter activities	FESV [32]	Continuous	4 to 20	x	x	x	x
Relaxation	FESV [32]	Continuous	4 to 20	x	x	x	x
Motivational self-efficacy	3 Items, based on [33]	Continuous	3 to 12	x	x		
Disorder and treatment knowledge	Own development	Continuous	0 to 50	x	x	x	x
Self-efficacy in practicing gained knowledge	Own development	Continuous	0 to 20	x	x	x	x
Electronic health literacy	eHEALS [34]	Continuous	8 to 40	x	x	x	x
Self-informing behaviour	Own development	Ordinal	1 to 6	x		x	x
Adherence to exercises	Own development	Ordinal	1 to 5			x	x
Adherence to knowledge	Own development	Ordinal	1 to 5			x	x
*Work functioning outcomes*							
Work ability in relation to work demands	Work Ability Index [35, 36]	Continuous	2 to 10	x	x	x	x
Self-rated work ability	Work Ability Index [37, 38]	Continuous	0 to 10	x	x	x	x
Current sickness absence	Own development	Binary		x	x	x	x
Sickness absence in weeks	Own development	Continuous	1 to 13 or 26	x		x	x
Employment	Own development	Binary		x		x	x
Employment contract	Own development	Nominal		x			
Shift working	Own development	Nominal		x			
*Patient satisfaction*							
Patient satisfaction	ZUF-8 [39]	Continuous	8 to 32		x		
System usability (intervention group only)	SUS [40]	Continuous	0 to 100		x		
Overall assessment of the Caspar application (intervention group only)	Based on Thielsch [41]	Ordinal	1 to 5		x		
Frequency of Caspar use (intervention group only)	Own development	Ordinal	1 to 6		x		
Type of electronic device (intervention group only)	Own development	Nominal			x		
*Rehabilitation aftercare*							
Physical activity	Own development	Nominal				x	x
Aftercare programs	Own development	Nominal				x	x
Reasons for non-use of aftercare	Own development	Nominal				x	x
**Other outcomes**							
Sociodemographic information	Own development	Various (year of birth, gender etc.)		x			
Treatments during the rehabilitation program	Standardized discharge report [42], according to the classification of therapeutic services [43]	Continuous (minutes or hours)			x		

### Primary outcome

The primary outcome is pain self-efficacy, which is measured by the German adaption of the Pain Self-Efficacy Questionnaire (PSEQ, German: Fragebogen zur Erfassung der schmerzspezifischen Selbstwirksamkeit, FESS) [25, 44]. The questionnaire consists of ten statements, which ask to what extent persons are convinced that they can carry out certain activities despite the pain. Accordingly, participants rate each statement on a scale from 1 to 6 (1 = completely convinced, 6 = not convinced at all). All scores are added up to an overall score (range from 10 to 60). Higher scores indicate better pain self-efficacy. Good validity of the FESS was shown in a rehabilitation setting [25].

### Secondary outcomes

*Health and health-related outcomes:* The current health status is assessed through one item of the COPSOQ (Copenhagen Psychosocial Questionnaire) [30]. The participants rate their current health condition on a scale from 0 to 10 (0 = worst imaginable state of health, 10 = best imaginable state of health).

Mental health, functional capacity, and pain are assessed with the IRES-24 questionnaire, which was developed as a comprehensive generic tool to measure outcomes of rehabilitation in Germany [31]. For mental health and functional capacity, eight questions or statements each are considered. Participants have five options to answer each question or statement (1 = usually, 2 = quite often, 3 = sometimes, 4 = rare, 5 = never). Pain is assessed using three questions with six options to answer (1 to 6 points). The mean scores of mental health, functional capacity, and pain are converted into a scale ranging from 0 to 10. Higher scores indicate better mental health or functional capacity and less pain.

Cognitive and behavioral pain management are determined by six subscores of the FESV questionnaire (Fragebogen zur Erfassung der Schmerzverarbeitung) [32]: action-oriented coping, cognitive restructuring, subjective coping competence, mental distraction, counter activities, relaxation. Each score is based on four statements. Participants rate each statement on a scale from 1 to 6 (1 = not true at all, 6 = absolutely true). The score of each statement is added up to an overall subscore ranging from 4 to 24, respectively. A higher overall score indicates a better outcome. The validity of the questionnaire was shown for a broad spectrum of pain patients [32].

Motivational self-efficacy is determined by ratings of three statements based on Schwarzer et al. [33]. Participants have four options to rate three statements (1 = it is not true, 2 = hardly true, 3 = rather true, 4 = true). The score of each question is added up to an overall score, which ranges from 3 to 12 points. A higher score shows better motivational self-efficacy.

Participants are expected to accumulate disorder and treatment knowledge on back pain during the rehabilitation. This knowledge is rated by ten self-developed questions (e.g., How well informed do you feel about the difference between specific and non-specific back pain?) A 6-point scale is provided for every question (0 = not informed at all, 5 = very good informed). The questions refer to all modules of the back school. The scores of the ten questions are added up to an overall score, which ranges from 0 to 50 points. A higher score indicates more disorder and treatment knowledge.

Participants are expected to improve their self-efficacy in practicing gained knowledge. Self-efficacy in practicing gained knowledge is tested by four self-developed questions (e.g., How confident do you feel in doing exercises to strengthen your back muscles?) A 6-point scale is provided for every question (0 = not confident at all, 5 = very confident). The questions are based on modules three and six of the back school. The scores of the four questions are added up to an overall score, which ranges from 0 to 20 points. A higher score indicates more self-efficacy in practicing gained knowledge.

Electronic health literacy is measured by the eHEALS (E Health Literacy Scale) [34]. Eight questions ask the participants how they rate their skills to obtain health-related information from the internet. Five choices to answer are provided for every question (1 = disagree, 2 = rather disagree, 3 = neutral, 4 = rather agree, 5 = totally agree). The score of each question is added up to an overall score. The overall score ranges from 8 to 40 points. A higher score is related to higher electronic health literacy.

Self-informing behavior is assessed using a self-developed question, which asks how frequently the participant informed themself about back pain or chronic pain in the past 3 months. Six potential answers are provided (1 = daily, 2 = several times per week, 3 = once per week, 4 = several times per month, 5 = rarely, 6 = never).

To assess how well the patients adhered to the exercises from the back school after the rehabilitation ended, we ask them the self- developed question: How often did you do the exercises from the back rehabilitation after the end of the rehabilitation? Five potential answers are provided (1 = several times a week, 2 = once per week, 3 = several times per month, 4 = rare, 5 = never).

To assess how well the patients adhered to the knowledge learned from the back school after rehabilitation ended, we ask them the self-constrcuted question: How often did you apply the knowledge you learned from the back rehabilitation to everyday life after the end of the rehabilitation? Five potential answers are provided (1 = several times a week, 2 = once per week, 3 = several times per month, 4 = rare, 5 = never).

*Work functioning outcomes:* Work ability is measured by three questions of the work ability index [35, 36]. The first and second question ask for the current ability to do physical work and mental work. For either question, five choices to answer are provided (5 = very good, 4 = rather good, 3 = moderate, 2 = rather bad, 1 = very bad). The score of the first two questions is added up to an overall score, which ranges from 2 to 10. A higher score is related to better work ability. Scoring is weighted by the type of work (mental, physical, or both mental and physical). A third question asks participants to rate their work ability from 0 to 10 points (0 = completely unable to work, 10 = best work ability) [37, 38].

Sickness absence is measured by self- developed questions, which assess if people are currently on sick leave and how many weeks patients were on sick leave during the last 6 months (baseline and 12-month follow-up) or 3 months (3-month follow-up).

Employment is assessed by a self- developed item, which allows a distinction between employed and non-employed persons. Additionally, we ask for the employment contract (e.g., permanent) and shift work.

*Patient satisfaction:* Patient satisfaction is assessed by the German version of the Client Satisfaction Questionnaire (CSQ, German: Fragebogen zur Messung der Patientenzufriedenheit, ZUF-8) [39, 45], which contains eight questions and four options to answer every question. After reversing four of the items, a total score is calculated ranging from 8 to 32 points. A higher score represents higher satisfaction.

*Use of the digital intervention:* The digital intervention is rated by the intervention group only. The system usability of the Caspar application is determined using the System Usability Scale (SUS) [40]. Participants rate ten statements on the utilization of the Caspar application by a 5-point scale (1 = strongly disagree, 5 = strongly agree). Negatively formulated questions are reversed before calculation. The sum of all 10 questions is calculated and multiplied by 2.5. The maximum value is 100, minimum value is 0.

An overall assessment of the Caspar application is assessed by grades from 1 to 5 (1 = very good, 2 = good, 3 = satisfying, 4 = sufficient, 5 = insufficient) [41].

The way of using the Caspar application is clarified by two self- developed questions. The first question asks how frequently participants make use of the Caspar application during the last 3 weeks. Six potential answers are provided (1 = daily, 2 = several times per week, 3 = once per week, 4 = several times per month, 5 = rarely, 6 = never). An additional question asks what electronic device was used. Multiple responses are possible (1 = laptop, 2 = computer, 3 = mobile phone, 4 = TV, 5 = tablet, 6 = other).

*Rehabilitation aftercare:* Three self- developed questions on rehabilitation aftercare assess which physical activity options were used in the last 3 months (including digital applications) (1 = rehabilitation sport, 2 = functional training, 3 = sports club, 4 = gym, strength endurance training or medical training therapy, 5 = endurance sports, e.g., running, Nordic walking, 6 = Yoga, Pilates, Qi Gong, or Tai Chi, 7 = I have not exercised in the last 3 months, 8 = other), which services were used in the last 3 months? (1 = none, 2 = T-RENA [exercise therapy rehabilitation aftercare], 3 = Caspar Health, 4 = social/vocational counseling, 5 = Psy-RENA [psychosomatic rehabilitation aftercare], 6 = occupational therapy, 7 = gym-based physiotherapy, 8 = IRENA [intensified rehabilitation aftercare], 9 = psychological counseling, 10 = functional training, 11 = physiotherapy, 12 = rehabilitation sport, 13 = other), and reasons for not participating in an aftercare program (1 = not provided by the outpatient rehabilitation center, 2 = no need, 3 = none of the rehabilitation aftercare programs around, 4 = no time for rehabilitation aftercare, 5 = no interest in rehabilitation aftercare, 6 = other). For each question multiple responses are possible.

### Other outcomes

Sociodemographic information on age, gender, language skills, partnership, number of children, level of education, and professional qualifications are collected by self- developed questions.

Therapeutic treatments during the rehabilitation program are recorded according to the classification of therapeutic services [43]. We derive these treatments and their duration in minutes or hours from the standardized medical discharge reports [42].

FESS: Pain Self-Efficacy Questionnaire (German: Fragebogen zur Erfassung der schmerzspezifischen Selbstwirksamkeit), COPSOQ: Copenhagen Psychosocial Questionnaire, IRES-24: Indicators of Rehabilitation Status (German: Indikatoren des Rehastatus), FESV: Questionnaire for the Assessment of Pain Processing (German: Fragebogen zur Erfassung der Schmerzverarbeitung), eHEALS: eHealth Literacy Scale, ZUF-8: Client Satisfaction Questionnaire (German: Fragebogen zur Messung der Patientenzufriedenheit), SUS: System Usability Scale. All instruments except the FESV were freely available and/or licenced with permission to use, share, adapt and reproduce. The FESV has to be purchased. Our self-developed items and questions can be used, shared, adapted and reproduced. Participant timeline.

A timeline of enrollment, intervention, and assessments is shown in Table 4.

**Table 4:** Participant timeline

*Timepoint*	*Pre-intervention*	*Beginning of intervention*	*End of intervention*	*3-month follow-up*	*12-month follow-up*
**Enrollment**					
Information and informed consent	x				
Randomization and allocation	x				
**Intervention**					
Rehabilitation		x	x		
**Assessments**					
Questionnaire		x	x	x	x
Standardized discharge report			x		
Guided interview			x	x	

### Sample size

**Fig. 1:**
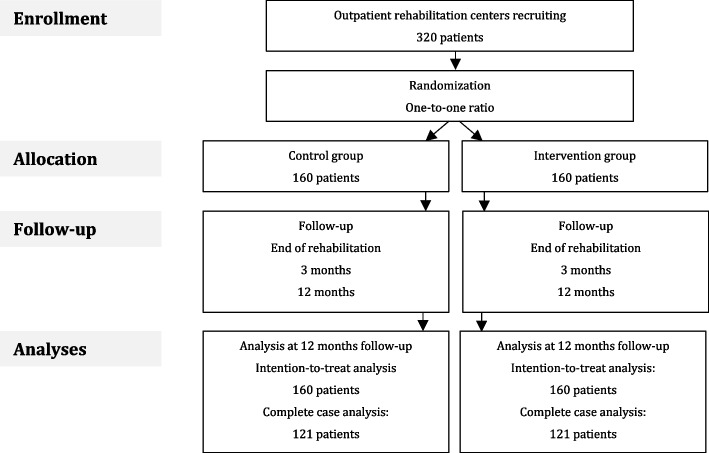
Flow of participants

A difference of four points for pain self-efficacy [25] was determined as the minimum relevant difference between the intervention and control group. The minimum required sample size for our non-inferiority analysis is 242 participants (one-sided error: 5%, power: 90%) to perform an intention-to-treat analysis after multiple imputation. The standard deviation for our sample size calculation was taken from the randomized controlled trial on the effectiveness of the study by Mangels et al. [46]. We are planning to recruit 320 participants from eight outpatient rehabilitation centers (intervention group: *n* = 160; control group: *n* = 160), which result in 20 participants for both arms of the study within each outpatient rehabilitation center. The sample size is sufficient for a secondary analysis including only patients with complete follow-up. The anticipated flow of participants is shown in Fig. 1.

### Recruitment

The recruitment is conducted in eight different outpatient rehabilitation centers. Study assistants recruit new study participants as follows: First, every new arriving patient is informed orally about the study. Second, eligible and interested study participants are handed a patient information leaflet as well as a consent form. Third, potential study participants are asked for their informed and written consent. The patient information letter and the consent form are accessible as additional file 3 and 4. Lastly, after informed consent, the study participant is randomly assigned to the intervention or control group.

### Allocation

Randomized allocation is done in a one-to-one ratio. The principal investigator at the University of Lübeck generated all randomization sequences using Stata 16.1 (StataCorp, College Station, Texas, USA). For each outpatient rehabilitation center, 40 assignments were randomly combined in blocks of four and eight, and the University of Lübeck provided the rehabilitation centers with 40 identical, non-transparent, sealed envelopes numbered from 1 to 40. The envelopes contain the information about the group allocation and personal identification number. Before opening the envelopes, the content of the envelopes was unknown to everyone, excluding the principal investigator. The envelopes are handed out consecutively from 1 upwards to the participants, after informed consent was given. A study assistant in each outpatient rehabilitation center enrolls the participants and registers the group allocation in the study list (Microsoft Excel) using the personal identification number. Due to the nature of the intervention, there is no blinding in the study.

### Data collection

Quantitative data are collected with questionnaires and standardized rehabilitation discharge reports. Qualitative data are collected with interviews by phone. An overview of the collected data and measurement time points is shown in Table 4. A description of the included assessments is given above.

The first and second questionnaires, together with a prepaid and preaddressed envelope to the University of Lübeck, are given to the participant by a study assistant in the outpatient rehabilitation center. Questionnaires are marked with the personal identification number of the participant only. The participant completes the questionnaires, puts them in the envelope and returns the sealed envelope to the study assistant. The study assistant sends the envelope to the University of Lübeck. For the third and fourth questionnaires, participants are contacted by mail through the University of Lübeck directly. Participants send the completed questionnaires back by again using a prepaid and preaddressed envelope. Patients whose follow-up questionnaires have not been received at the University of Lübeck two weeks after completion are reminded once by mail with new questionnaires, as well as a prepaid and preaddressed envelope to the University of Lübeck. The standardized medical discharge reports are sent to the University of Lübeck after the recruitment phase is over and contain all documented therapeutic treatments during rehabilitation. Researchers from the University of Lübeck conduct the guided interviews between the second and third measurement time points. Participants can withdraw their consent at any time and all collected data will be deleted accordingly.

### Data management

Data from the questionnaires are entered into computers by trained research assistants or researchers from the University of Lübeck using Microsoft Access forms. The Microsoft Access forms contain the exact set of questions and values from the questionnaires. Data entry is done directly after the questionnaires arrive at the University of Lübeck.

Documented in the study list of each outpatient rehabilitation center is whether the first and second questionnaires were given to the participant, the personal identification number, name, surname, year of birth, address, and gender, as well as the start and end date of rehabilitation. The study list is transferred to the University of Lübeck.

In the monitoring list at the University of Lübeck, all study lists are combined and received questionnaires from all participants at all measurement time points are documented. It is also documented whether the third and fourth questionnaires were sent to the participant from the University of Lübeck, and if written consent was received. Additionally, the University of Lübeck uses the monitoring list is to send out reminder letters to the participants in case of missing questionnaires.

### Data monitoring

An external data monitoring committee is not implemented. Short-term and long-term effects are analyzed and published separately. No further interim analyses are planned. No criteria for early study termination have been established.

### Auditing

Every 2 weeks, the status of recruitment, response rates of the questionnaires, other announcements, and current events are discussed through a video conference between the University of Lübeck and the outpatient rehabilitation centers.

### Confidentiality

Every new study participant is entered into the study list at each outpatient rehabilitation center. All documentation is done by study assistants from the outpatient rehabilitation centers. The study list is password protected. This list is stored on a computer at the outpatient rehabilitation center, and only the study assistants have access to it. Every two weeks, the study assistants send an electronic encrypted copy of the study list to the researchers at the University of Lübeck. After the last enrolled participant finishes the second questionnaire, the study assistants delete the study list.

All received study lists from the outpatient rehabilitation centers are incorporated into the monitoring list at the University of Lübeck. Each received study list is deleted immediately at the University of Lübeck after the content is transferred to the monitoring list. The monitoring list is password protected and stored on the computers at the University of Lübeck. Only researchers involved in the study have access to it. The monitoring list itself will be deleted after the third follow-up is complete (anonymization of the data).

The data from all questionnaires are pseudonymized by the personal identification number on the questionnaire. All completed questionnaires are sent to the University of Lübeck. At the University of Lübeck, the questionnaires are stored in folders and locked in lockers that belong to the office rooms of researchers who are involved in the study. The questionnaires are only accessible to researchers who are involved in the study. All questionnaires will be destroyed in accordance with data protection regulations upon completion of the study.

The interviews are recorded and stored on the computers at the University of Lübeck. The files of the audio recordings are password protected and pseudonymized immediately after the interview is finished. Transcripts of the interviews are also pseudonymized. The audio recordings are deleted after the interviews are transcribed.

Medical discharge reports are pseudonymized by the personal identification number and sent electronically to the University of Lübeck by the study assistant at each outpatient rehabilitation center.

All digital data is stored on password protected computers that require authentication of users. The computers are located in the researchers’ office rooms at the University of Lübeck, which are locked when left. Access to the computers can be tracked. Only researchers at the University of Lübeck have access to the digital data. The network in which the computers are integrated is protected against external access and manipulation by a regularly updated firewall system.

Ten years after the end of the study, all digitalized data from the questionnaires will be deleted.

### Access to data

All authors of the study protocol will have access to the final and fully anonymized data set.

### Statistical analysis

We analyze the short-term effects (end of rehabilitation and 3-months follow-up) and long-term effects (12-months follow-up) to show non-inferiority of the hybrid rehabilitation program. The results of the short- and long-term effects will be published separately.

To test the primary hypothesis of non-inferiority, the confidence interval method is applied [47, 48]. We determined -4 points as the non-inferiority margin. Four points are slightly below the smallest clinically important difference (5.5 to 8.5 points) reported in a recent systematic review on pain self-efficacy in patients with low back pain [49]. We assume non-inferiority of the hybrid rehabilitation if the lower boundary of the one-sided 95% confidence interval (CI) exceeds -4 points.

The estimate of the treatment effect is adjusted for the baseline scores of the dependent variable, as well as for the treatment center (outpatient rehabilitation center). To estimate group differences for continuous and binary outcome variables, linear or logistic regression is calculated. Regression coefficients or odds ratios and their 95% CI are determined. Confidence intervals are two-sided for all secondary outcomes.

Age, sex, level of education, electronic health literacy, and motivational self-efficacy are considered as moderators of the treatment effect on our primary outcome variable. The interaction effects are assessed through p-values. All secondary outcome variables are tested on superiority of the intervention group, and we present p-values. We test superiority to identify potential group differences that, in the case of non-inferiority, can be used to formulate a preference for one of the two treatments [50].

In order to perform an intention-to-treat analysis, missing values are imputed by multiple imputation using 20 independent data sets [51, 52]. The parameter estimates are combined according to Rubin’s rules [53].

A sensitivity analysis also calculates unadjusted estimates for the intention-to-treat analysis. An additional sensitivity analysis is conducted as complete case analysis. Like in the primary analysis, we adjust for the baseline scores of the dependent variable and for the treatment center. We also conduct a per-protocol analysis, including only individuals in both groups who participated in six of the seven back school modules.

Withdrawal from consent leads to deletion of the participant’s data.

The analyses are performed with Stata 16.1 (StataCorp, College Station, Texas, USA). A two-tailed p-value of less than 0.05 is considered statistically significant.

## Discussion

Our randomized controlled trial compares a hybrid rehabilitation that implements an online back school with a rehabilitation that conventionally provides a face-to-face back school. We aim to demonstrate non-inferiority of the hybrid rehabilitation. All findings of this study will be published in peer-reviewed journals and presented at conferences. The authors of this present study protocol will also write the publication of the findings. No professional writers will be included in the process.

The SPIRIT checklist (Standard Protocol Items: Recommendations for Interventional Trials) was used when drafting the study protocol [54].

### Trial status

Recruitment has started and is still ongoing.

## Supplementary Information

**Additional file 1.** Items from the World Health Organization Trial Registration Data Set.

**Additional file 2. **Full description of the intervention according to the TIDieR checklist and the TIDieR-telehealth checklist.

**Additional file 3. **Patient information.

**Additional file 4. **Model consent form.

**Additional file 5. **Self-developed instruments.

## Data Availability

The datasets used and/or analyzed during the current study are available from the corresponding author on reasonable request.

## Abbreviations

FESS: Pain Self-Efficacy Questionnaire (German: Fragebogen zur Erfassung der schmerzspezifischen Selbstwirksamkeit)
COPSOQ: Copenhagen Psychosocial Questionnaire
IRES-24: Indicators of Rehabilitation Status (German: Indikatoren des Rehastatus)
FESV: Questionnaire for the Assessment of Pain Processing (German: Fragebogen zur Erfassung der Schmerzverarbeitung)
eHEALS: EHealth Literacy Scale
ZUF-8: Client Satisfaction Questionnaire (German: Fragebogen zur Messung der Patientenzufriedenheit)
SUS: System Usability Scale
SPIRIT: Standard Protocol Items: Recommendations for Interventional Trials
TIDieR: Template for Intervention Description and Replication
HCP: Health care professional
