# Supplementary material for: Non-inferiority of a hybrid outpatient rehabilitation: a randomized controlled trial (HIRE, DRKS00028770)
Source: BMC Digit Health. 2023 Apr 25;1(1):15. doi: 10.1186/s44247-023-00013-4 (PMC10125254; doi:10.1186/s44247-023-00013-4)
Supplement: Supplementary file 1 — Additional file 1. Items from the World Health Organization Trial Registration Data Set. [file 44247_2023_13_MOESM1_ESM.docx]

Table S1: Items from the World Health Organization Trial Registration Data Set

| **Data category** | **Information** |
| --- | --- |
| Register | German Clinical Trials Register |
| Last refreshed on | 23/01/2023 |
| Main ID | DRKS00028770 |
| Date of registration | 05/04/2022 |
| Prospective registration | Yes |
| Primary sponsor | Universität zu Lübeck, Institut für Sozialmedizin und Epidemiologie |
| Public title | Non-inferiority of a hybrid outpatient rehabilitation: A randomized controlled trial |
| Scientific title | Non-inferiority of a hybrid outpatient rehabilitation: A randomized controlled trial (HIRE) |
| Date of first enrolment | 19/04/2022 |
| Target sample size | 320 |
| Recruitment status | Ongoing |
| URL | https:// www.drks.de/drks_web/navigate.do?navigationId=trial.HTML&TRIAL_ID=DRKS00028770 |
| Study type | Interventional |
| Allocation | Randomized controlled trial |
| Masking | Open (masking not used) |
| Control | Control group receives treatment as usual |
| Assignment | Parallel |
| Purpose | Treatment |
| Phase | N/A |
| Countries of recruitment | Germany |
| Contacts | Stella Lemke Ratzeburger Allee 160, 23562 Lübeck, Germany +49 451 50051282 [stella.lemke@uksh.de](mailto:stella.lemke@uksh.de)  Universität zu Lübeck, Institut für Sozialmedizin und Epidemiologie |
| Key inclusion and exclusion criteria | Inclusion criteria: Patients with back pain are included (ICD-10 M50–M54)  Exclusion criteria: Patients without a stable Internet connection, an appropriate electronic device for watching videos via browser or app, a suitable camera for communication or sufficient German language skills are excluded  Age minimum: 18 years  Age maximum: 65 years  Gender: Both male and female |
| Health conditions or problems studied | ICD-10: M50–M54 – other dorsopathies |

| Interventions | Intervention 1: The participants in the intervention group are prescribed a standardized back school, implemented digitally using the Caspar application. The back school contains seven modules (Module 1: basics; Module 2: back health and movement behaviour; Module 3: body perception and spinal column stabilization; Module 4: psychological factors; Module 5: posture and movement sequences in everyday life and at work; Module 6: physical activity in everyday life: Part 1; Module 7: physical activity in everyday life: Part 2). All modules are completed during a 3-week rehabilitation program. The modules consist of three elements: education, physical exercises and interactive online meetings. Educational content and physical exercises are accessible individually through videos and take 45 minutes for completion per module. Experienced physical therapists in telemedicine will guide the interactive online meetings. Every patient takes part in one interactive online meeting per week (45 minutes). Personal electronic devices of the patients, such as smartphones or laptops, are used to follow the modules or to contact the physical therapists via chat, which means the participants are not tied to a specific location. The digital content is available at any time.  The patients follow additional individual rehabilitation programs over the day in every outpatient rehabilitation center during the 3-week rehabilitation (e.g. endurance or relaxation training). This multimodal rehabilitation program follows the therapy standard for back pain rehabilitation as recommended by the German Pension Insurance. Therefore, the term “hybrid rehabilitation” is appropriately used for the intervention group.  Intervention 2: The participants in the control group also receive the seven modules of the standardized back school, which is a standard component of 3-week back pain rehabilitation in Germany and is provided in face-to-face meetings. Just as in the intervention group, the modules consist of education, physical exercises and interactive elements. Completion of each module takes 60 minutes. Experienced health experts (e.g. physical therapists, psychologists, doctors) guide the face-to-face meetings. Only analogue materials are used.  The patients follow additional individual rehabilitation programs over the day in every outpatient rehabilitation center during the 3-week rehabilitation (e.g. endurance or relaxation training). This multimodal rehabilitation program follows the therapy standard for back pain rehabilitation as recommended by the German Pension Insurance. |
| --- | --- |
| Primary outcome | The primary outcome is pain self-efficacy, which is measured by the FESS (Fragebogen zur Erfassung der schmerzspezifischen Selbstwirksamkeit) questionnaire, the German adaption of the Pain Self-Efficacy Questionnaire (PSEQ). A total score is calculated, with a higher value representing a greater degree of pain self-efficacy (range 10–60) (Mangels et al., 2009). It is measured at all four measurement time points: start of rehabilitation (T1), end of rehabilitation (T2), 3 months after the end of rehabilitation (T3) and 12 months after the end of rehabilitation (T4). |
| Secondary outcomes | Current health status is measured continuously from 0 to 10 by one item of the COPSOQ (Nübling et al., 2005). Measurement time points: T1, T2, T3, T4.  Mental health is measured continuously from 0 to 10 by the IRES-24 (Wirtz et al., 2005). Measurement time points: T1, T2, T3, T4.  Functional capacity is measured continuously from 0 to 10 by the IRES-24 (Wirtz et al., 2005). Measurement time points: T1, T2, T3, T4.  Pain is measured continuously from 0 to 10 by the IRES-24 (Wirtz et al., 2005). Measurement time points: T1, T2, T3, T4.  Action-oriented coping is measured continuously from 4 to 20 by the FESV (Geissner, 1999). Measurement time points: T1, T2, T3, T4.  Cognitive restructuring is measured continuously from 4 to 20 by the FESV (Geissner, 1999). Measurement time points: T1, T2, T3, T4.  Subjective coping competence is measured continuously from 4 to 20 by the FESV (Geissner, 1999). Measurement time points: T1, T2, T3, T4.  Mental distraction is measured continuously from 4 to 20 by the FESV (Geissner, 1999). Measurement time points: T1, T2, T3, T4.  Counter-activities are measured continuously from 4 to 20 by the FESV (Geissner, 1999). Measurement time points: T1, T2, T3, T4.  Relaxation is measured continuously from 4 to 20 by the FESV (Geissner, 1999). Measurement time points: T1, T2, T3, T4.  Motivational self-efficacy is measured continuously from 3 to 12 based on Schwarzer et al. (2007). Measurement time points: T1, T2.  Disorder and treatment knowledge is measured continuously from 0 to 50 (own construction). Measurement time points: T1, T2, T3, T4.  Self-efficacy in practicing gained knowledge is measured continuously from 0 to 20 (own construction). Measurement time points: T1, T2, T3, T4.  Electronic health literacy is measured continuously from 8 to 40 by the eHEALS (Norman & Skinner, 2007). Measurement time points: T1, T2, T3, T4.  Self-informing behavior is measured ordinally with categories from 1 to 6 (own construction). Measurement time points: T3, T4.  Adherence to exercises is measured ordinally with categories from 1 to 5 (own construction). Measurement time points: T3, T4.  Adherence to knowledge is measured ordinally with categories from 1 to 5 (own construction). Measurement time points: T3, T4.  Work ability in relation to work demands is measured continuously from 2 to 10 by three items of the WAI (Ilmarinen, 2007). Measurement time points: T1, T2, T3, T4.  Self-rated work ability is measured continuously from 0 to 10 by one item of the WAI (Ilmarinen, 2007). Measurement time points: T1, T2, T3, T4.  Current sickness absence is measured using a binary yes/no (own construction). Measurement time points: T1, T2, T3, T4.  Sickness absence during the last 6 months is measured using a binary yes/no (own construction). Measurement time points: T1, T4.  Sickness absence during the last 3 months is measured using a binary yes/no (own construction). Measurement time points: T3.  Sickness absence in weeks is measured continuously from 1 to 13 or 26 (own construction). Measurement time points: T1, T3, T4.  Employment is measured using a binary yes/no (own construction). Measurement time points: T1, T3, T4.  Employment contract is measured nominally (own construction). Measurement time points: T1.  Shift working is measured nominally (own construction). Measurement time points: T1.  Patient satisfaction is measured continuously from 8 to 32 by the ZUF-8 (Attkisson & Zwick, 1982). Measurement time points: T2.  System usability is measured continuously from 0 to 100 by the SUS (Brooke, 1986). Measurement time points: T2 (intervention group only).  Overall assessment of the Caspar application is measured ordinally with categories from 1 to 5 (Thielsch & Salaschek, 2017). Measurement time points: T2 (intervention group only).  Frequency of Caspar use is measured ordinally with categories from 1 to 6 (own construction). Measurement time points: T2 (intervention group only).  Type of electronic device is measured nominally (own construction). Measurement time points: T2 (intervention group only).  Physical activity is measured nominally (own construction). Measurement time points: T3, T4.  Aftercare programs are measured nominally (own construction). Measurement time points: T3, T4.  Reasons for non-use of aftercare are measured nominally (own construction). Measurement time points: T3, T4.  Sociodemographic data: year of birth, gender, language skills, partnership, number of children, level of education and professional qualifications. Measurement time points: T1.  Treatments during the rehabilitation program: coding will be done according to the Classification of Therapeutic Services Report (Deutsche Rentenversicherung, 2015) and Standardized Medical Discharge Report (Deutsche Rentenversicherung, 2014). Measurement time point: T2. |
| Secondary ID(s) | 21-462 (Ethics Committee, University of Lübeck)  U1111-1264-9325 (Universal Trial Number) |
| Source of monetary support | Federal German Pension Insurance (Deutsche Rentenversicherung Bund) |
| Status of ethics review | Approved |
| Approval date of ethics review | 25/03/2022 |
