# Supplementary material for: Non-inferiority of a hybrid outpatient rehabilitation: a randomized controlled trial (HIRE, DRKS00028770)
Source: BMC Digit Health. 2023 Apr 25;1(1):15. doi: 10.1186/s44247-023-00013-4 (PMC10125254; doi:10.1186/s44247-023-00013-4)
Supplement: Supplementary file 2 — Additional file 2. Full description of the intervention according to the TIDieR checklist and the TIDieR-telehealth checklist. [file 44247_2023_13_MOESM2_ESM.docx]

Table S2: Full description of the intervention group based on the TIDieR checklist and the TIDieR-Telehealth checklist

| **Brief name** | Digital back school (hybrid rehabilitation) |
| --- | --- |
| **Why** | The back school aims to increase physical activity in everyday life by teaching knowledge about the development and maintenance of back pain, communicating a positive functional image of the back, demonstrating and practicing exercises to strengthen the back, reflecting how pain is mentally processed and promoting the transfer of physical activity in everyday life [1]. Telerehabilitation and hybrid rehabilitation may improve the accessibility of rehabilitative services and the application of behaviors learned in rehabilitation to everyday life. |
| **What (materials)** | All content of the back school is digitalized and accessible online, via app or web browser, using the Caspar application. Patients use their private electronic device to participate. A study assistant within each outpatient rehabilitation center gives oral and practical instructions on correct use of the Caspar application and hands out a booklet with further information on the program, including the individual login account of the patient. Videos on physical exercises and educational videos cover all seven modules of the back school. Patients can use this multimedia content at any time and place. Only participants from the intervention group have access to the digitalized back school. Videos for physical exercises provide several options. The patient can choose between different modes of intensity, velocity and forms of performance. A self-view is optional through the camera of the electronic device in order to correct the body movements during the exercise. Each module contains one educational video, which is up to 14 minutes long and can be watched as often as wanted. Moreover, a chat box provides an interactive element for questions from the patients and feedback by a health care professional (HCP), in particular physiotherapists. Patients can use this chat box at any time during the week. For further interactive meetings, the electronic device must be equipped with a suitable camera. Interactive online meetings are implemented through a video conference tool. In order to participate in these meetings, the patients receive personal login details via email, or they can scan a QR code that leads directly to the online meeting. The QR code is handed out as a one-pager after randomization. After completion of a module, the patient completes a short quiz (using the previously mentioned booklet), which serves as a learning check and as a tool for educational reflection. It also informs the HCP of the level of activity in the back school. The answers to the completed quiz can be uploaded in the chat box. |
| **What (procedures)** | The back school consists of seven modules [2]: (1) fundamentals (information about back pain and therapies); (2) back health and physical activity (structure and function of the spine, body awareness, pain and physical activity); (3) body awareness and spine stabilization (muscle activation strategies to stabilize the spine and loosening exercises); (4) mental factors (pain behavior and coping strategies); (5) posture and movement sequences in everyday life and at work (appropriate sitting, bending, lifting, carrying with muscle active spine stabilization at home and work); (6) physical activity in everyday life: Part 1 (motivation and intention); and (7) physical activity in everyday life: Part 2 (action and coping planning). More details on the content of the modules can be found elsewhere [2]. |
| **Who provided** | Caspar Health is a private company located in Berlin (Germany) that is specialized in digital rehabilitation applications and digital patient care. Different experts (e.g. healthcare or movement scientists, psychologists, physicians, physical and occupational therapists) are involved in their products. Caspar Health provides all features of the Caspar application, including all multimedia content (e.g. videos on physical exercises or the causes of back pain). They also ensure that the Caspar application reflects the same content of the modules as the standard therapy. For that purpose, various experts are included in the production.  The Tele-Therapie Klinik Berlin, which is based in Berlin (Germany), is a clinic in telemedicine and it provides all the HCPs that guide the interactive sessions in the intervention group online, including the chat. These HCPs are experienced and specialized in digital therapy. The Tele-Therapie Klinik Berlin plans, organizes and schedules all the interactive meetings. |
| **How** | The entire rehabilitation program is implemented online using the Caspar application. Group interactive meetings are performed by an HCP via camera. Non-interactive parts of the modules are performed by each patient independently. |
| **Where** | The patients participate in the intervention from home. The HCPs operate from the buildings of Tele-Therapie Klinik Berlin or from home. |
| **When and how much** | The seven modules are completed within 3 weeks. Each module requires 45 minutes to complete. The patients can choose freely when to use the educational videos and the videos on physical exercises during the week. In addition, every week a live interactive meeting is conducted online via camera and a video conference tool for 45 minutes. Interactive meetings are scheduled during the afternoon. The participants can choose from different time slots.  Week 1: Modules 1 and 2 include educational videos and videos on physical exercises (45 minutes per module) via the Caspar application and one interactive meeting via Zoom (45 minutes), including a discussion as well as an introduction and reflection/quiz on the modules.  Week 2: Modules 3–5 include educational videos and videos on physical exercises (45 minutes per module) via the Caspar application and one interactive meeting via Zoom (45 minutes), including a discussion as well as an introduction and reflection/quiz on the modules.  Week 3: Modules 6 and 7 include educational videos and videos on physical exercises (45 minutes per module) via the Caspar application and one interactive meeting via Zoom (45 minutes), including a discussion as well as an introduction and reflection/quiz on the modules.  In addition to the back school, the patients follow their individual 3-week rehabilitation program over the day in the outpatient rehabilitation centers. Treatments of the rehabilitation programs follow the therapy standards developed by the Federal German Pension Insurance for the rehabilitation of chronic back pain [3]. |
| **Tailoring** | No specific tailoring of the intervention to the patient is planned. Nevertheless, during the weekly interactive meeting and in the chat, patients can receive individual feedback from the performing HCP and apply it accordingly (e.g. change a physical exercise if painful). |
| **Modifications** | Not applicable. |
| **How well** | No strategies are implemented to maintain adherence to rehabilitation. However, it will be registered if and how often the patients watched the videos and if patients joined the weekly interactive meetings. Therapy adherence will be measured and assessed by the researchers of the University of Lübeck. |

Table S3: Full description of the control group based on the TIDieR checklist and the TIDieR-Telehealth checklist

| **Brief name** | Face-to-face back school (conventional rehabilitation) |
| --- | --- |
| **Why** | The back school aims to increase physical activity in everyday life by teaching knowledge about the development and maintenance of back pain, communicating a positive functional image of the back, demonstrating and practicing exercises to strengthen the back, reflecting how pain is mentally processed and promoting the transfer of physical activity in everyday life [1]. |
| **What (materials)** | Module 1: beamer, presentation sheets, spine of a skeleton, patient information booklet (if applicable).  Module 2: skeleton, charts, flip chart, patient information booklet (if applicable).  Module 3: standing desk, lordosis cushion, seat wedge, ball cushion, gymnastic ball, stool, gymnastic mattresses, patient information booklet (if applicable).  Module 4: overhead projector, presentation sheets, flip chart, work sheet within the patient information booklet (if applicable), pencils (for the patients).  Module 5: therapy couches, ball, stool, box with little sand bags for additional weight, flip chart, patient information booklet (if applicable).  Module 6: flip chart, work and presentation sheets, posters, patient information booklet (if applicable), pencils (for the patients).  Module 7: flip chart, work and presentation sheets, posters, work sheet within patient information booklet (if applicable), pencils (for the patients).  The patient information booklet provides information on all seven modules (educational information, physical exercises). A more detailed description on the usage of all materials can be found elsewhere [2]. |
| **What (procedures)** | The back school consists of seven modules [2]: (1) fundamentals (information about back pain and therapies); (2) back health and physical activity (structure and function of the spine, body awareness, pain and physical activity); (3) body awareness and spine stabilization (muscle activation strategies to stabilize the spine and loosening exercises); (4) mental factors (pain behavior and coping strategies); (5) posture and movement sequences in everyday life and at work (appropriate sitting, bending, lifting, carrying with muscle active spine stabilization at home and work); (6) physical activity in everyday life: Part 1 (motivation and intention); and (7) physical activity in everyday life: Part 2 (action and coping planning). More details on the content of the modules can be found elsewhere [2]. |
| **Who provided** | The face-to-face back school has been provided at outpatient rehabilitation centers of the Nanz medico GmbH & Co. KG since 1996 as part of a 3-week rehabilitation program. The back school is provided by a physician (Module 1), a physical therapist (Modules 2, 3, 5–7) and a psychologist (Module 4). The back school is implemented and scheduled by each outpatient rehabilitation center individually. |
| **How** | The back school is carried out in groups within the outpatient rehabilitation center via face-to-face meetings (10–20 people per meeting). Outside of the meetings patients can make use of their materials, such as the patient information booklet, in order to practice or do the exercises. |
| **Where** | All meetings take place in the outpatient rehabilitation center. |
| **When and how much** | The seven modules are completed within 3 weeks. Each module requires 60 minutes to complete. In addition to the back school, the patients follow their individual 3-week rehabilitation program over the day in the outpatient rehabilitation center. The treatments of the rehabilitation programs are in accordance with the therapy standards developed by the German Pension Insurance for the rehabilitation of chronic back pain [3]. |
| **Tailoring** | No specific tailoring of the intervention to the patient is planned. During the meetings, patients can receive individual feedback from the performing physiotherapist and apply it accordingly (e.g. change a physical exercise if painful). |
| **Modifications** | As all included outpatient rehabilitation centers are separate institutions, slight variations in performing the standardized back school may occur. |
| **How well** | No strategies are implemented to maintain adherence to rehabilitation. However, it will be registered if patients joined each module or not. Therapy adherence will be measured and assessed by the researchers of the University of Lübeck. |

**References**

1. Meng K, Seekatz B, Rossband H, Worringen U, Faller H et al. Development of a standardized back training program for orthopedic rehabilitation [German]. *Rehabilitation* 2009; 48: 335-344.

2. Hoppe K, Oehme J, Worringen U. *Curriculum Back School: Standardized Patient Training* [German]. Berlin: Deutsche Rentenversicherung Bund; 2019.

3. Deutsche Rentenversicherung*.* *Rehabilitation Therapy Standards for Chronic Back Pain* [German]. Berlin: Deutsche Rentenversicherung Bund; 2020.
