# Supplementary material for: Non-inferiority of a hybrid outpatient rehabilitation: a randomized controlled trial (HIRE, DRKS00028770)
Source: BMC Digit Health. 2023 Apr 25;1(1):15. doi: 10.1186/s44247-023-00013-4 (PMC10125254; doi:10.1186/s44247-023-00013-4)
Supplement: Supplementary file 3 — Additional file 3. Patient information. [file 44247_2023_13_MOESM3_ESM.docx]

## Information

**on participation in our study ‘HIRE – non-inferiority of a hybrid outpatient orthopedic rehabilitation’**

Dear patient,

This patient information informs you about our study ‘Non-inferiority of a hybrid outpatient orthopedic rehabilitation’ (HIRE). The Institute for Social Medicine and Epidemiology at the University of Lübeck is conducting the study, together with your outpatient rehabilitation center (ZAR). Based on the following information, you can decide whether to participate in our study. You can keep this information letter.

#### What is the study about?

First, what is hybrid rehabilitation? Hybrid rehabilitation means that one part of the rehabilitation is implemented directly at your rehabilitation center and another part of the rehabilitation takes place digitally at home (courses from the back school). In these digital units at home, the rehabilitants take part in group and individual sessions virtually and are guided by a professional rehabilitation team. You can participate in this digital rehabilitation via an Internet-enabled device.

There will be two groups in this study. One group will participate in the hybrid rehabilitation and another group will participate regularly in the standard rehabilitation at the rehabilitation center. By comparing the two groups, we can determine if hybrid and standard rehabilitation have different or similar effects on the patients. Allocation to either of the groups is at random and is called randomization.

Why is it important? Digital or telerehabilitative therapy measures are becoming increasingly important in the field of rehabilitation and open up the opportunity for more flexible implementation of rehabilitation. The study we are planning compares hybrid rehabilitation involving digital seminars with rehabilitation in which the seminars are conducted conventionally. This study is funded by the Federal German Pension Insurance (Deutsche Rentenversicherung Bund).

Prof. Dr. Matthias Bethge from the Institute for Social Medicine and Epidemiology at the University of Lübeck is the principal investigator.

#### Which questions do the researchers clarify?

The study is planned in an outpatient orthopedic rehabilitation setting. In addition to the regular treatment options, one part of the seminars will be provided digitally. The researchers seek to clarify how this new treatment option is implemented and to what extent it is comparable with regular care in outpatient rehabilitation.

#### What will my pension insurance provider find out?

The pension insurance provider does not receive any information about your participation or non-participation.

#### How is the study planned?

The staff at our rehabilitation center have already checked whether you are eligible to take part in the study and that is why we have approached you. With this letter you will receive a questionnaire and a declaration of consent. In order to be able to determine the effect of hybrid rehabilitation compared to standard rehabilitation, we ask you to fill out a total of four questionnaires.

The first questionnaire should be completed now, at the beginning of your rehabilitation. You will receive the second questionnaire at the end of your rehabilitation and the third and fourth questionnaires will be sent to you 3 and 12 months, respectively, after the end of your rehabilitation.

All four questionnaires give us information about the long-term development of your state of health. We keep the questionnaires for as short a time as possible but we are also interested in forming an accurate idea of your care. In the four questionnaires we will ask you about your self-efficacy, health, work ability and participation. We would also like to know how you rate the rehabilitation.

In addition to the information you provide in our questionnaires, we would like to assess other data from your medical records that is relevant for our study, specifically, a list of the diagnostic and therapeutic services you received. The documentation assistant at the rehabilitation center collects the necessary data from the medical records, pseudonymizes it (the process of pseudonymization is explained in the following section) and then sends it via email to the researchers at the University of Lübeck.

In addition, if you were to be selected by us, we would be happy to ask you directly about your experiences via a telephone interview.

#### How do we deal with the collected data?

If you have given your consent to participate in our study, your name and address will be entered into a study list by your rehabilitation center and you will be assigned a study number. This study number will be written on your questionnaires. The data from your medical records will also be provided with this study number. This procedure is called pseudonymization and ensures that we cannot draw any conclusions about the participants from the research data. Basically, your name does not appear on the questionnaires or in the data from your medical reports! By using the study number, the researchers can link the questionnaire data with the data from the medical records. This is very important to us because we care about the outcome of the treatment. The list of participants will be destroyed by your rehabilitation center at the end of the study, which means that the names and addresses of the study participants will no longer be available (anonymization).

We researchers receive a copy of the study list for postal mailing of the questionnaires 3 and 12 months after the end of the rehabilitation. This list may not be used by us for any purpose other than for sending the questionnaires. The list is stored separately from the research data collected, is protected by a password and will be destroyed immediately after the last questionnaires have been sent. In this way, we ensure that no one can unnecessarily link the research data with your name. Nevertheless, we may only collect the data with your explicit, voluntary and written consent, and all researchers are obliged to treat your data confidentially.

All personal statements in the interviews will be anonymized when they are written down, so that it is no longer possible to draw any conclusions about the participants. Afterwards, the data will be evaluated. The tape recordings are also deleted after they have been written down. The transcripts will be stored on password-protected computers at the University of Lübeck for the duration of the study and will be destroyed in accordance with data protection regulations no later than 10 years after the end of the study.

#### Participation and your rights

Participation in the study is voluntary. If you decide not to participate, you will not suffer any disadvantages. You can also withdraw your participation at a later date. If you would like to revoke your participation, receive information about your stored data or exercise your right to data deletion, please contact Richard Albers at the University of Lübeck (email: [richardmaurice.albers@uksh.de](mailto:richardmaurice.albers@uksh.de)).

At this point we would like to inform you about your rights.

*Right to information (Article 15 data protection regulation):* You have the right to information about the personal data stored about you.

*Right to rectification (Article 16 data protection regulation):* If you find that incorrect data about you is being processed, you can request correction. Incomplete data must be completed by taking into account the purpose of the processing.

*Right to erasure (Article 17 data protection regulation):* You have the right to request the deletion of your data if there are certain reasons for deletion. This is particularly the case if it is no longer required for the purpose for which it was originally collected or processed.

*Right to restriction of processing (Article 18 data protection regulation):* You have the right to restrict the processing of your data. This means that although your data will not be erased, it will be marked to restrict any further processing or use.

*Data portability (Article 20 data protection regulation):* If the processing is carried out using an automated procedure based on your consent, you have the right to request that your data be provided in a structured, common and machine-readable format.

*Right to object to unreasonable data processing (Article 21 data protection regulation):* In principle, you have a general right to object to data processing.

*Right to lodge a complaint with a supervisory authority (Article 77 data protection regulation):* You have the right to lodge a complaint with a supervisory authority if you believe that the processing of your personal data violates the data protection regulation.

#### Responsible institutions

The Institute for Social Medicine and Epidemiology at the University of Lübeck is responsible for evaluating the anonymous data and supervising the implementation of the study.

Responsibility for the study lies with Prof. Dr. Matthias Bethge at the University of Lübeck (email: [matthias.bethge@uksh.de](mailto:matthias.bethge@uksh.de)).

| Data protection officer: University of Lübeck  x-tention Informationstechnologie GmbH  Karl-Drais-Str. 4e  86167 Augsburg  Germany  Phone: +49 451 3101 1903  Email: datenschutz(at)uni-luebeck(dot)de | Supervisory authority: University of Lübeck  State Facility for Data Protection Schleswig-Holstein  Holstenstraße 98  24103 Kiel  Germany |
| --- | --- |

#### We kindly ask you to participate

The success of the study depends on a large number of participants. The more people who take part, the more meaningful the results will be. We therefore ask you to decide whether to participate. If you have any further questions, we will be happy to address them.

We look forward to your participation and thank you in advance for your help.

With kind regards,

|  | N. N.  University of Lübeck  Institute for Social Medicine and Epidemiology  Tel.: +49 451 xxxxxxxx  Email: n.n@nnnn.de |
| --- | --- |
