# Supplementary material for: Non-inferiority of a hybrid outpatient rehabilitation: a randomized controlled trial (HIRE, DRKS00028770)
Source: BMC Digit Health. 2023 Apr 25;1(1):15. doi: 10.1186/s44247-023-00013-4 (PMC10125254; doi:10.1186/s44247-023-00013-4)
Supplement: Supplementary file 4 — Additional file 4. Model consent form. [file 44247_2023_13_MOESM4_ESM.docx]

## Declaration of consent

**on participation in our study ‘HIRE – non-inferiority of a hybrid outpatient orthopedic rehabilitation’**

Mrs./Mr. ………………………………………… …………………………………………

Name Surname

………………………………..……………………………….....

Phone number (optional)

I have been informed about the content and purpose of the HIRE study, which is funded by the Federal German Pension Insurance (Deutsche Rentenversicherung Bund). Prof. Dr. Matthias Bethge from the University of Lübeck is responsible for the study. I was given information on participation in the study, including information on data protection.

I am willing to support the HIRE study through my participation and give consent to (please mark applicable options below).

⭘ Fill in the provided questionnaires.

⭘ Take part in a guided interview by telephone, if selected.

⭘ Beyond that, I consent to give access to data in my medical records during the time of rehabilitation. The type of required data is described in the letter of information on participation.

I consent to my rehabilitation center extracting the relevant information for this purpose from the medical records and providing this information via a study identification number to the researchers of the University of Lübeck. For transmission of personal data from my medical records to the researchers of the University of Lübeck, I release the clinic staff from their medical confidentiality. The release from medical confidentiality refers exclusively to the data collection as part of the HIRE study.

I have been informed about my data protection rights. I consent to the collection, processing, transmission and storage of my data, as part of the study.

I consent to the outpatient rehabilitation center (ZAR) passing on my contact details (name, telephone number, email address, address) to the researchers at the University of Lübeck so that they can send me the follow-up questionnaires (reminder and third/fourth questionnaires) and to schedule an interview. This personal data is used exclusively to send the follow-up questionnaires and will be deleted after sending, in accordance with data protection regulations.

I was comprehensively informed about my rights, but also about the time required as a study participant.

With my signature, I declare my consent to participate in the study under the conditions described in the letter of information on participation.

Place, date: …………………………..………….. Signature: …………………..………………..………………..
