# Supplementary material for: Non-inferiority of a hybrid outpatient rehabilitation: a randomized controlled trial (HIRE, DRKS00028770)
Source: BMC Digit Health. 2023 Apr 25;1(1):15. doi: 10.1186/s44247-023-00013-4 (PMC10125254; doi:10.1186/s44247-023-00013-4)
Supplement: Supplementary file 5 — Additional file 5. Self-developed instruments. [file 44247_2023_13_MOESM5_ESM.docx]

**Additional file 5: Self-developed instruments**

# Health and related outcomes

## Motivational self-efficacy

Sometimes things turn out differently than planned. How sure are you that you will be able to do the following after completing rehab?

I am sure…

1. … that I can be physically active at least once a week.
2. … that I can be physically active for twenty minutes at least twice a week.
3. … that I can change my life to a physically active lifestyle.

Response options: ‘Not true’, ‘Hardly true’, ‘Rather true’, ‘True’.

## Disorder and treatment knowledge

The following is about the extent to which you are informed about back pain.

1. How well informed do you feel about the difference between specific and nonspecific back pain?
2. How well informed do you feel about risk factors for the development and progression of back pain?
3. How well informed do you feel about different treatment methods for back pain?
4. How well informed do you feel about the positive effects of movement?
5. How well informed do you feel about assistive devices (e.g., assistive devices at your workplace)?
6. How well do you feel informed about biological, psychological, and social factors that may influence the experience of pain?
7. How well informed do you feel about the biological, psychological, and social consequences of back pain?
8. How well informed do you feel about the difference between acute and chronic pain?
9. How well informed do you feel about appropriate exercise and sports for back pain?
10. How well informed do you feel about ways to monitor your actions in achieving your exercise goals (e.g., exercise log)?

Response options: 0 = not informed at all, 5 = very well informed.

## Self-efficacy in practicing gained knowledge

The following is about how confident you feel in implementing exercises to stabilize the back and with personal movement goals.

1. How confident do you feel in performing exercises to strengthen your back muscles?
2. How confident do you feel in performing loosening exercises?
3. How confident do you feel in planning your own physical activity goals?
4. How confident do you feel in dealing with barriers you may encounter in achieving your physical activity goals?

Response options: 0 = not at all confident, 5 = very confident.

## Self-informing behaviour

In the past three months, how often did you inform yourself about back pain or chronic pain? (e.g., information on causes of back pain or chronic pain; measures to treat back pain or chronic pain).

Response options: ‘Daily’, ‘Several times a week’, ‘Once a week’, ‘Several times a month’, ‘Rare’, ‘Never’.

## Adherence to exercise

How often did you perform the exercises from the rehabilitation after rehab ended?

Response options: ‘Several times a week’, ‘Once a week’, ‘Several times a month’, ‘Rare’, ‘Never’.

## Adherence to knowledge

How often did you apply the knowledge you learned from the rehabilitation in your everyday life after the end of rehab?

Response options: ‘Several times a week’, ‘Once a week’, ‘Several times a month’, ‘Rare’, ‘Never’.

# Work functioning outcomes

## Current sickness absence

Are you currently on sick leave (unable to work)?

Response options: ‘Yes’, ‘No’.

## Sickness absence in weeks

Have you been on sick leave (unable to work) in the last 6* months? If yes, how many weeks?

Please round up to whole weeks (e.g. 17 days = 3 weeks).

*Or ‘…in the last 3 months’ in the second questionnaire.

Response options: ‘Yes’, ‘No’. If ‘yes’, how long (in weeks)?

## Employment status

Which statement describes your current employment status best?

1. Employed full-time with 35 hours or more per week.
2. Employed part-time with 15 to 34 hours per week.
3. Part-time or hourly employed with less than 15 hours per week.
4. In training/education.
5. Not employed.
6. Other, please specify: …

## Employment contract

What is your current work situation? Are you...

1. employed with a permanent contract?
2. employed with a fixed-term contract?
3. employed with a permanent contract with a temporary employment agency?
4. employed with a temporary contract with a temporary employment agency?
5. Other: …

## Shift working

Do you work in shifts?

1. No.
2. Yes, I work in 2 shifts.
3. Yes, I work in 3 shifts or more.

# Patient satisfaction

## Overall assessment of the Caspar application

All in all: I give Caspar (website or app) the overall grade ...

1. 1 (very good)
2. 2 (good)
3. 3 (satisfying)
4. 4 (sufficient)
5. 5 (insufficient)

## Frequency of Caspar use

With which device did you (mainly) use Caspar? Multiple answers possible.

1. Laptop
2. Computer
3. Mobile phone
4. TV
5. Tablet
6. Other, please specify: …

## Type of electronic device

How often have you used Caspar in the past three weeks?

1. Daily
2. Several times per week
3. Once per week
4. Several times per month
5. Rarely
6. Never

# Rehabilitation aftercare

## Physical activity

What physical activity options have you used in the past 3 months (including digital options)? Multiple responses are possible.

1. Rehabilitation sport
2. Functional training
3. Sports club
4. Gym, strength endurance training or medical training therapy
5. Endurance sports, e.g., running, Nordic walking, cycling, swimming, fast walking.
6. Yoga, Pilates, Qi Gong, or Tai Chi
7. I have not exercised in the last 3 months
8. Other, please specify: …

## Aftercare programs

In the last 3 months, have you used any of the following services to ensure rehab success and follow-up care? Multiple responses are possible.

1. None
2. T-RENA
3. Caspar Health
4. Social/vocational counseling
5. Psy-RENA
6. Occupational Therapy
7. Gym-based physiotherapy
8. IRENA
9. Psychological counselling
10. Functional Training
11. Rehabilitation sport
12. Other, please specify: …

## Reasons for non-use of aftercare

If you did not used any of the rehab aftercare services or programs: Why did you not use any of the services listed in question 16? Multiple responses are possible.

1. It was not available at the rehab clinic.
2. There was no need.
3. None of the services were close to where I live.
4. No time for rehab aftercare.
5. No interest in rehab aftercare.
6. Other, please specify: …

# Sociodemographic information

## Gender

Are you:

1. Female
2. Male
3. Other

## Year of birth

What year were you born?

## Language

Is German your first language?

Response options: ‘Yes’, ‘No’.

## Partnership

Do you have a permanent partnership?

Response options: ‘Yes’, ‘No’.

## Number of children

Do you have children?

1. No
2. 1 child
3. 2 children
4. 3 children
5. More than 3 children

## Level of education

What is your highest school-leaving qualification? Please make one cross only.

1. Secondary general school [Germany: level 2 of the ISCED-2011]
2. Intermediate school [Germany: level 2 of the ISCED-2011]
3. Intermediate school in the German Democratic Republic [Germany: level 2 of the ISCED-2011]
4. Specialized upper secondary school [Germany: level 3 of the ISCED-2011]
5. Upper secondary school [Germany: level 3 of the ISCED-2011]
6. Other school leaving certificate [Germany: if not proven differently, level 2 of the ISCED-2011]
7. No school leaving certificate [Germany: level 1 of the ISCED-2011]

## Professional qualifications

What is your highest professional qualification? Please make only one cross.

1. Vocational training [Germany: level 3 or level 4 of the ISCED-2011]
2. Trade and technical schools (e.g. master craftsman’s diploma; university of cooperative education) [Germany: level 6 of the ISCED-2011]
3. College; university of applied sciences [Germany: level 6 of the ISCED-2011]
4. University [Germany: level 7 of the ISCED-2011]
5. In training/education [Germany: level 1, level 2 or level 3 of the ISCED-2011]
6. No professional qualification [Germany: level 1, level 2 or level 3 of the ISCED-2011]
